# Polymorphisms in the adrenergic neurotransmission pathway impact antidepressant response in depressed patients

**DOI:** 10.1016/j.nsa.2022.101016

**Published:** 2022-11-18

**Authors:** Taichi Ochi, Natalya M. Vyalova, Innokentiy S. Losenkov, Diana Z. Paderina, Ivan V. Pozhidaev, Anton J.M. Loonen, German G. Simutkin, Nikolay A. Bokhan, Bob Wilffert, Svetlana A. Ivanova

**Affiliations:** a Department of PharmacoTherapy, -Epidemiology and -Economics, Groningen Research Institute of Pharmacy, University of Groningen, Antonius Deusinglaan 1, 9713AV, Groningen, the Netherlands; b Mental Health Research Institute, Tomsk National Research Medical Center of the Russian Academy of Sciences, Aleutskaya str., 4, Tomsk, 634014, Russian Federation; c Department of Psychotherapy and Psychological Counseling, National Research Tomsk State University, Lenin Ave., 36, Tomsk, 634050, Russian Federation; d Department of Psychiatry, Addictology and Psychotherapy, Siberian State Medical University, Mos-kovsky trakt, 2, Tomsk, 634050, Russian Federation; e Department of Clinical Pharmacy and Pharmacology, University Medical Center Groningen, University of Groningen, Hanzeplein 1, 9713 GZ, Groningen, the Netherlands; f School of Non-Destructive Testing and Security, Division for Control and Diagnostics, National Research Tomsk Polytechnic University, Lenin Avenue, 30, 634050, Tomsk, Russian Federation

**Keywords:** Major depressive disorder, Antidepressants, Treatment response, Adrenergic neurotransmission, Pharmacogenetics, Habenula

## Abstract

Mood disorders are a prevalent mental health disorder. The adrenergic neurotransmission pathway presents an opportunity to determine whether genetic mutations impact antidepressant response. For this study, 163 patients with major depressive disorders were enrolled to measure treatment response using the Hamilton Depression Rating Scale (HAMD-17). More than half of the patients had never been treated with antidepressants previously. Patients were genotyped for 14 SNPs within *ADRA1A*, *SLC6A2*, *ADRβ1*, *MAOA* and *COMT* to determine the impact of adrenergic neurotransmission polymorphisms related in antidepressant response. Patients were treated mainly with SSRIs and TCAs. The difference in HAMD-17 scores between the measurement periods were defined as the outcome measure. Multiple linear regression was conducted to determine the association between the genotypes and difference in HAMD-17 across the study period. Covariates of age, sex, antidepressant medication and depression diagnoses were included in the regression. Throughout the study HAMD-17 scores were measured at initiation, at two weeks and at four weeks for each patient. The difference in HAMD-17 scores was found to be 11.2 ​± ​4.4 between initiation and two weeks, 7.8 ​± ​5.3 between two week and four week, and 19.0 ​± ​5.3 throughout the entire study. *SLC6A2* rs1532701 homozygous G/G Patients were associated with improved ΔHAMD-17 across week 2–4 and the entire study (B ​= ​7.1, p ​= ​0.002; B ​= ​6.7, p ​= ​0.013) compared to homozygous A/A patients. *SLC6A2* rs1532701 homozygous A/G patients were further associated with improved ΔHAMD-17 compared to homozygous A/A patients at week 2–4 (B ​= ​2.8, p ​= ​0.023). Through our investigation, we were able to determine the genes within the adrenergic pathway to investigate further. To further elucidate these findings, replication and combination with other neurotransmitter pathways to better map the mechanism of actions of antidepressant for tailored treatment would be suggested.

## Highlights

• A classic depression theory postulates that antidepressants act by increasing adrenergic neurotransmission.
• Functional genetic variants must then influence the response to adrenergic antidepressants.
• *SLC6A2* polymorphisms (NET) associated with the varied response to antidepressants.
• Dopamine D4 receptors in the habenula are stimulated by norepinephrine from adrenergic neurons.
• Possibly the habenula mediates the antidepressant action of adrenergic antidepressants.

## 1 Introduction

Depressive mood disorders are one of the most common mental health problems. They occur not only in mood disorders in the strict sense - such as major depressive disorder (MDD), bipolar depression and dysthymia - but also in patients with psychotic disorders such as schizophrenia or personality disorders (Möller, 2005; Doherty et al., 2014). In the classical view, the MDD is described as a delineated disorder with a specific pathogenetic basis that, incidentally, is likely more complex than those found in infectious diseases. Multiple theories have been put forward to explain the origins of the MDD (Loonen and Ivanova, 2016b). The monoamine theory is the oldest of these and within the framework of the theory, it was first thought a dysregulation of adrenergic neurotransmission was a possible cause for MDD (Schildkraut, 1965; Coppen, 1967; Maas, 1979). This theory was initially based on the influence of substances with therapeutic effects in the treatment of MDD (Loonen and Ivanova, 2016b) and - although this theory is now highly debated - raising cerebral concentrations of norepinephrine (NE), 5-hydroxytryptamine (5-HT) and dopamine (DA) continues to be considered an important target site for antidepressant drugs (Hamon and Blier, 2013).

Our group has recently developed a neuronal model for the development of mood disorders (Loonen and Ivanova, 2016a, 2018). Ascending adrenergic pathways, which use norepinephrine as a neurotransmitter, play an important role in this. Fibers originating in the locus coeruleus complex run to the shell part of the nucleus accumbens (NAcbS) and to the (frontal) cerebral cortex; these fibers use beta-receptors in their synapses (Loonen and Ivanova, 2016b). Fibers running from other adrenergic nuclear regions to the hypothalamus use alpha-adrenoceptors. With the use of adrenergic antidepressants, these receptors are acutely more strongly stimulated by inhibiting the reuptake or inhibiting the breakdown of norepinephrine. This can be accomplished by inhibiting the norepinephrine transporter (NET) or monoamine oxidase type A (MAO-A). Incidentally, inhibition of NET also results in stronger stimulation of dopamine receptors (Ochi et al., 2021) and the breakdown of both norepinephrine and dopamine is also mediated by catechol-O-methyl transferase (COMT) (Westfall et al., 2018). In addition to these interactions at the neurochemical level, the many neuronal interactions between the adrenergic, serotonergic and dopaminergic neurotransmitter systems should also be mentioned (Hamon and Blier, 2013; Hensler et al., 2013).

We have recently reported on studies targeting genetically determined variants of proteins involved in serotonergic neurotransmission (Ochi et al., 2019) and dopaminergic receptors and enzymes (Ochi et al., 2021) in the magnitude of the effects of antidepressant drugs. For this current investigation, we looked to determine whether genetic variants of adrenergic neurotransmission genes would impact antidepressant response.

## 2 Experimental procedures

### 2.1 Patient characteristics

The study design and patient characteristics have been described in previous publications (Ochi et al., 2019). Newly admitted patients with a depressive episode according to the criteria of ICD-10 (F32 or F33) (World Health Organization, 2004) who had not been on antidepressant medication for at least 6 months were recruited. More than half the patients have never been treated with antidepressant medication during their entire life. An increased number of patients were genotyped compared to previous studies, as an additional expansion of the scope of the study.

The patients' depression was of at least moderate severity as measured by the Hamilton's depression rating scale (HAMD-17) (Hamilton, 1960; Williams et al., 1992). Moderate depression in HAMD-17 is categorised by a score of 18–23. After this initial examination, an antidepressant treatment of at least four weeks was initiated and patients were re-examined with HAMD-17 after two and four weeks. Details pertaining to the inclusion and exclusion criteria were outlined previously (Ochi et al., 2019). The study complied with the Declaration of Helsinki (1975, revised in Fortaleza, Brazil, 2013) and was submitted and authorized by the Ethics Committee of the Mental Health Research Institute, Tomsk National Research Medical Center (protocol 49 from 23.04.12). All patients were recruited from psychiatric departments of this institute and provided written informed consent.

### 2.2 Genotyping

Blood samples were drawn from antecubital venepuncture in evacuated EDTA tubes and aliquots were stored at −20 ​°C. DNA was isolated from leucocytes using the standard phenol-chloroform micro method. Polymorphisms in genes pertaining to the adrenergic pathway were genotyped in the Laboratory of Genetics of the University of Groningen with the MassARRAY® System (Agena Bioscience™) and in the Laboratory of Molecular Genetics and Biochemistry of the Mental Health Research Institute with “StepOnePlus” (Applied Biosystems). The full list of SNPs can be found in Supplementary Table 1.

### 2.3 Selection of genotypes

A post hoc power calculation was conducted to determine the number of investigated SNPs. 14 candidate SNPs were selected from the 51 sequenced variants. The selected SNPs met the Hardy–Weinberg equilibrium, had a minor allele frequency (MAF) of >5%, and had been reported to be associated with neurological or psychiatric disorders (schizophrenia, alcoholism, autism, Parkinson's disease) for further analysis. These polymorphisms were localised within *ADRA1A* (rs2036108), *ADRB1* (rs1801253), *COMT* (rs4680, rs6269, rs4633, rs4818, rs165774), *MAOA* (rs6323, rs1137070) and *SLC6A2* (rs2242446, rs36024, rs1532701, rs13333066, rs187714) genes.

### 2.4 Statistical analysis

To determine the effect of adrenergic pathway genes to antidepressant response, the outcome was measured by the difference in HAMD-17 score between entry and two weeks of treatment (ΔHAMD-17, 0–2 weeks) after two and four weeks of treatment (ΔHAMD-17, 2–4 weeks) and entry and four weeks of treatment (ΔHAMD-17, 0–4 weeks). Normal distribution was tested utilizing the P–P plot.

Patients characteristics, including age, sex, HAMD-17 scores across the different study periods, were emphasised using descriptive statistics. Univariate linear regression was conducted to determine the SNPs to include in the multiple linear regression. Multiple linear regression was conducted to identify the independent factors associated with ΔHAMD-17 between the three time periods, including age, sex, depression diagnosis, type of antidepressant taken and selected SNPs. To factor in the different categories in antidepressant taken and SNP genotypes, dummy variables were generated to establish the effect in each variable. Statistical analysis was conducted with SPSS software (release 25.0). The significance level for descriptive statistics and univariate statistical tests were p ​< ​0.05. Factoring in Bonferroni correction, the significance level for multiple linear regression was p ​< ​0.0031. Power analysis was conducted post-hoc utilizing G∗Power.

## 3 Results

Summary statistics of the cohort, genotypes investigated and antidepressant medication are outlined in Table 1, Supplementary Tables 2 and 3 The study population was mainly women, with 141 participants compared 22 males (86.5% vs 13.5%). Most patients took SSRIs (n ​= ​100), specifically sertraline (n ​= ​26), paroxetine (n ​= ​23), escitalopram (n ​= ​17), fluoxetine (n ​= ​14) and fluvoxamine (n ​= ​12).

**Table 1:** Patient characteristics.

Characteristics	Depressed Cohort (n ​= ​163)
Total Number of Patients (%)	
Male	22 (13.5%)
Female	141 (86.5%)
Age in Years (Mean ​± ​S.D.)	49.5 ​± ​10.9
Male	49.3 ​± ​9.3
Female	49.5 ​± ​11.1
HAMD-17 score (Mean ​± ​S.D.)	
At entry	24.1 ​± ​4.9
At 2 weeks	12.9 ​± ​5.0
At 4 weeks	5.1 ​± ​3.9
Type of Depressive Episode (%)	
Single	92 (56.4%)
Recurrent	71 (43.6%)

Comparing the medication taken, ΔHAMD-17 was significantly more improved in participants taking tricyclic antidepressants at 0–2 weeks and 0–4 weeks (B ​= ​2.9, p ​= ​0.004; B ​= ​4.4, p ​= ​0.0007, respectively) (Table 2, Supplementary Table 3). Patients who were *SLC6A2* rs1532701 homozygous G/G were associated with improved ΔHAMD-17 across week 2–4 and the entire study (B ​= ​7.1, p ​= ​0.002; B ​= ​6.7, p ​= ​0.013) (Supplementary Table 4) when compared to homozygous A patients. *SLC6A2* rs1532701 homozygous A/G patients were further associated with improved ΔHAMD-17 compared to homozygous A/A patients at week 2–4 (B ​= ​2.8, p ​= ​0.023). Patients carrying the minor alleles in *COMT* SNPs (rs4680, rs6269, rs4633, rs4818) were found at 0–2 weeks with significant ΔHAMD-17 scores, however in polar extremes.

**Table 2:** Multiple linear regression of total depression cohort covariates (age, gender, diagnosis, type of antidepressant, selected adrenergic genotypes) for the whole study period (0–4 weeks).

Baseline Predictors	B	95% CI	p-value	Baseline Predictors	B	95% CI	p-value
(Constant)	17.46	11.68–23.25					
Age	1.57	−1.1 - 4.23	0.25				
Gender	0.01	−0.08–0.09	0.86				
Diagnosis	−1.49	−3.38–0.39	0.12				
ADRA1A SNPs				COMT SNPs			
rs2036108 ​G/A	0.14	−1.78–2.06	0.89	rs4680 ​G/A	0.36	−8.93–9.65	0.94
rs2036108 A/A	−1.24	−6.49–4.01	0.65	rs4680 A/A	−8.76	−23.43–5.91	0.24
				rs6269 A/G	−2.81	−9.6–3.99	0.42
SLC6A2 SNPs				rs6269 ​G/G	−0.34	−23.21–13.23	0.59
rs2242446 ​T/C	−0.03	−5.27–5.21	0.99	rs4633 ​C/T	−0.28	−9.17–8.61	0.96
rs2242446 ​C/C	−3.93	−9.98–2.12	0.21	rs4633 ​T/T	8.58	−5.89–23.05	0.25
rs36024 ​T/C	−1.69	−4.75–1.37	0.28	rs4818 ​C/G	1.73	−4.78–8.25	0.60
rs36024 ​C/C	4.04	−1.52–9.6	0.16	rs4818 ​G/G	7.16	−10.45–24.76	0.43
rs1532701 A/G	1.97	−0.82–4.76	0.17	rs165774 ​G/A	−0.34	−2.44–1.77	0.75
rs1532701 ​G/G	6.65	1.43–11.88	0.013∗	rs165774 A/A	−1.68	−5.16–1.81	0.35
rs13333066 ​C/T	1.37	−3.55–6.28	0.59				
rs13333066 ​T/T	−2.52	−10.01 - 4.96	0.51	SLC6A3 SNPs			
rs187714 ​C/T	−0.35	−3.18–2.48	0.81	rs1801253 ​C/G	−0.61	−2.51–1.29	0.53
rs187714 ​T/T	−5.49	−10.42–−0.57	0.03	rs1801253 ​G/G	−2.26	−9.14–4.61	0.52
MAOA SNPs				Treatment (compared to SSRIs)			
rs6323 TG	0.46	−4.75–5.66	0.87	TCAs	4.40	2.04–6.77	0.0007∗∗∗
rs6323 GG	−4.81	−14.63 - 5	0.34	SNRIs	0.35	−2.75–3.44	0.83
rs1137070 CT	0.17	−5.03 - 5.36	0.95	NaSSAs	−1.40	−4.99–2.19	0.45
rs1137070 TT	5.36	−3.91–14.63	0.26	Agomelatine	0.88	−2.56–4.33	0.62
						R-squared	
						0.28	

Data is presented as regression coefficients (B), 95% confidence intervals (CI) and total explained variance (r2); ∗p ​< ​0.05; Significance for p values after correction: ∗∗p ​< ​0.0031; ∗∗∗p ​< ​0.001; HAMD, Hamilton Depression Score Rating Difference; TCAs, tricyclic antidepressants; SNRIs, serotonin–norepinephrine reuptake inhibitors; NaSSAs, noradrenergic and specific serotonergic antidepressants.

## 4 Discussion

Following our previous investigations on polymorphisms impacting serotonergic and dopaminergic neurotransmissions, this investigation looked to determine the impact of polymorphisms in adrenergic receptors and enzymes on antidepressant response in our study cohort. Considering our study cohort, we matched the number of variants studied to the resulting statistical power resulting from this sample size via Power Analysis. Therefore, we estimate that the statistical associations found for the minor alleles of *COMT* are incidental due to the Bonferroni correction to mitigate multiple testing. As the responses difference in patients were found to be in the opposite directions, the sum value of *COMT* polymorphisms would not be indicative of antidepressant response. Previously, COMT SNP rs4680, was associated with predicting antidepressant response but we were not able to corroborate the findings here (Baune and Arolt, 2006).

**Fig. 1:**
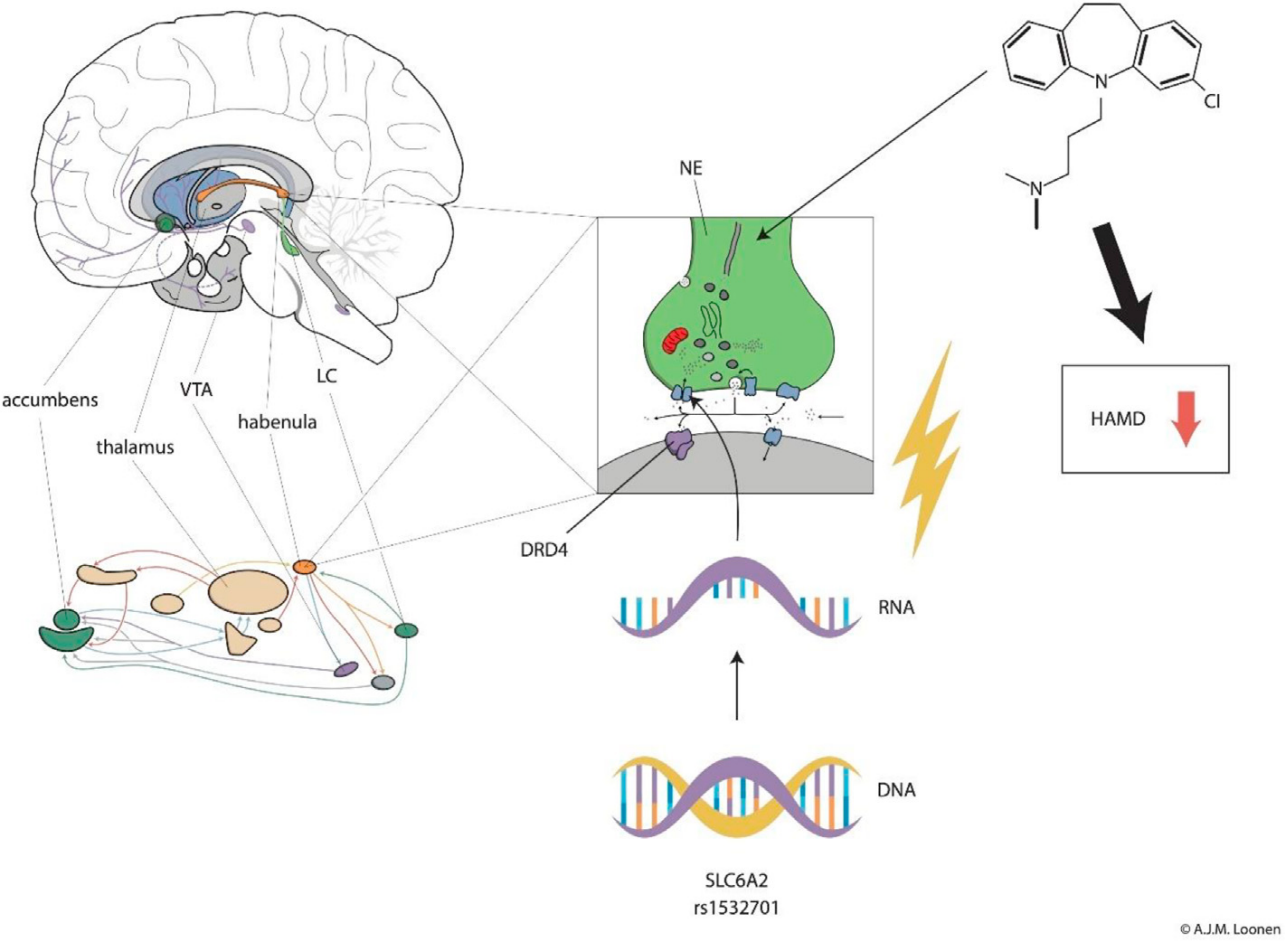
Figure demonstrating the possible role of the habenula The figure above shows the regulation of the activity of the secondary forebrain, with the dorsal diencephalic connection system (via the habenula) directing the activity of the ascending monoaminergic pathways. The adrenergic locus coeruleus (LC) is also connected to the habenula. This circuit is shown in the central block, where adrenergic LC neurons activate postsynaptic dopamine D4 receptors. By inhibiting norepinephrine reuptake, clomipramine (top right) brings about a decrease in the HAMD score *i.e.* has an additional antidepressant effect. *SLC6A2* rs1532701 is denoted from the findings of this investigation as an example of polymorphisms potentially impacting antidepressant response. DRD4: dopamine D4 receptor; HAMD: Hamilton Depression Rating Scale 17; LC: locus coeruleus; SCL6A2: gene encoding norepinephrine transporter (NET); VTA: ventral tegmental area.

As we consider each genetic polymorphism separately for our hypothesis-driven research, the application of the Bonferroni correction is warranted. From our investigation, *SLC6A2* rs1532701 polymorphisms were associated with changes in antidepressant response. Interestingly, *SLC6A2* rs1532701 has been previously associated with venlafaxine reduction (Yeh et al., 2015) but within the Chinese Han population. No further studies have found a strong association with the polymorphism, bringing the discussion of whether the polymorphism may be ethnic specific. The most striking finding is the existence of a significant association with the magnitude of the response to antidepressants and the possession of some genetic variants of the *SLC6A2* encoding NET protein. This comes as no big surprise, as NET is considered one of the two main targets of antidepressants. In our previous study, we found evidence of an association of response with dopamine D4 receptor polymorphisms (Ochi et al., 2021). It is worth mentioning that in the habenula these receptors are stimulated by norepinephrine released from adrenergic fibres (Root et al., 2015). Dopamine D4 receptors have quite high affinity for NE (Cummings et al., 2010; Newman-Tancredi et al., 1997). This leads us to our hypothesis, which suggests that adrenergic antidepressants may come into action by influencing dopamine D4 receptors in the habenula. The habenula plays an important role in our model of the emergence of mental disorders (Loonen and Ivanova, 2019). This evolutionarily very old part of the epithalamus regulates the activity of ascending monoaminergic pathways of the midbrain (Fig. 1). In turn, this affects the activities of ventral extrapyramidal re-entry circuits that are responsible for the intensity of reward-seeking and distress-avoiding behaviours (Loonen and Ivanova, 2016a, 2018, 2019).

Whereas this study cohort demonstrated antidepressant response by the participants, our investigation did not delver further into the response per individual. At the initiation of our study, we included participants that were categorised with at least moderate depression (HAM-D ​> ​18). However, while the mean HAM-D of treatment fell during the four weeks of the study within the Not Depressed (HAM-D 0–7) and Subthreshold Depression (8–13) range, not all patients were fully responsive to the antidepressant treatment. Among the non-responders, we had two participants that still had a HAM-D greater than 18. Non-response to antidepressant treatment is, unfortunately, a common enough occurrence that it impedes optimal treatment management in patients (Papakostas et al., 2020). Nevertheless, through our investigation of adrenergic loci, we endeavoured to better understand the mechanisms in which treatment could be optimised in depressed patients.

Due to the small size of the study, our adrenergic loci genes must be considered preliminary. However, we find that they are of sufficient interest to further investigate the significance of dopamine D4 receptors in the lateral habenula for the mechanism of action of antidepressant treatments. For future studies, our aim would be to focus on expanding the findings from the different neurotransmitter studies towards developing a interlinked pathway to determine the optimal SNPs to investigate for tailoring antidepressant treatment.

## Role of the funding source

This research received no external funding

## Contributors

Tachi Ochi contributed to the methodology, validation, formal analysis, writing – original draft preparation, writing – review and editing, visualization; Natalya M. Vyalova contributed to the investigation, writing – review and editing; Innokentiy S. Losenkov contributed to the investigation, writing – review and editing; Diana Z. Paderina contributed to the investigation; Ivan V. Pozhidaev contributed to the investigation; Anton J.M. Loonen contributed to the conceptualization, methodology, validation, writing – original draft preparation, writing – review and editing, visualization, supervision, funding acquisition; German G. Simutkin contributed to the investigation, writing – review and editing, supervision; Nikolay A. Bokhan contributed to the resources, supervision; Bob Wilffert contributed to the resources, supervision, project administration; Svetlana A. Ivanova contributed to the conceptualization, methodology, validation, resources, data curation, writing – review and editing, supervision, project administration.

## Declaration of competing interest

The authors declare that they have no known competing financial interests or personal relationships that could have appeared to influence the work reported in this paper.
