# Supplementary material for: Polymorphisms in the adrenergic neurotransmission pathway impact antidepressant response in depressed patients
Source: Neurosci Appl. 2022 Nov 18;2:101016. doi: 10.1016/j.nsa.2022.101016 (PMC12243976; doi:10.1016/j.nsa.2022.101016)
Supplement: Multimedia component 1 [file mmc1.docx]

**Supplementary material**

**Supplementary Table 1** – Investigated adrenergic genes

| Gene | Chromosome | Locus | SNP | Alleles |
| --- | --- | --- | --- | --- |
| ADRA1A | 8 | p21.2 | rs2036108 | GA/AA |
|  |  |  |  |  |
| SLC6A2 | 16 | q12.2 | rs2242446 | TC/CC |
|  |  |  | rs36024 | CT/TT |
|  |  |  | rs1532701 | GA/AA |
|  |  |  | rs13333066 | CT/TT |
|  |  |  | rs187714 | CT/TT |
|  |  |  |  |  |
| MAOA | X | p11.3 | rs6323 | GT/GG |
|  |  |  | rs1137070 | CT/TT |
|  |  |  |  |  |
| COMT | 22 | q11.21 | rs4680 | AG/AA |
|  |  |  | rs6269 | GA/GG |
|  |  |  | rs4633 | TC/TT |
|  |  |  | rs4818 | GC/GG |
|  |  |  | rs165774 | AG/AA |
|  |  |  |  |  |
| ADRB1 | 10 | q25.3 | rs1801253 | CG/GG |

**Supplementary Table 2** – Antidepressant medication taken by patients

| Class | Antidepressant | Patients |
| --- | --- | --- |
| SSRIs | Total | 100 |
|  | Sertraline | 26 |
|  | Paroxetine | 23 |
|  | Escitalopram | 17 |
|  | Fluoxetine | 14 |
|  | Fluvoxamine | 12 |
|  | Citalopram | 4 |
|  | Trazodone + Paroxetine | 2 |
|  | Fluvoxamine + Agomelatine | 1 |
|  | Trazodone | 1 |
| TCAs | Total | 25 |
|  | Clomipramine | 18 |
|  | Pipofezine | 6 |
|  | Amitriptyline | 1 |
| SNRIs | Total | 15 |
|  | Venlafaxine | 11 |
|  | Duloxetine | 4 |
| Agomelatine | Agomelatine | 12 |
| NaSSa | Total | 11 |
|  | Mirtazapine | 7 |
|  | Mianserin | 4 |

Antidepressant taken over the course of the four weeks by patients. SSRIs: selective serotonin receptor inhibitors, TCAs: tricyclic antidepressants; SNRIs: serotonin–norepinephrine reuptake inhibitors; NaSSAs: noradrenergic and specific serotonergic antidepressants.

**Supplementary Table 3** - Multiple linear regression of total depression cohort covariates (age, gender, diagnosis, type of antidepressant, selected adrenergic genotypes) for the first two week study period (0 to 2 weeks).

| Baseline Predictors | | B | 95% CI | p-value | Baseline Predictors | | B | 95% CI | p-value |
| --- | --- | --- | --- | --- | --- | --- | --- | --- | --- |
| (Constant) | | 14.62 | 9.76 - 19.48 |  |  |  |  |  |  |
| Age | | -0.92 | -3.16 - 1.32 | 0.42 |  |  |  |  |  |
| Gender | | -0.04 | -0.11 - 0.04 | 0.32 |  |  |  |  |  |
| Diagnosis | | -1.46 | -3.04 - 0.13 | 0.08 |  |  |  |  |  |
|  |  |  |  |  |  |  |  |  |  |
| ADRA1A SNPs | |  |  |  | COMT SNPs | |  |  |  |
|  | rs2036108 G/A | -0.46 | -2.08 - 1.15 | 0.57 |  | rs4680 G/A | -5.78 | -13.58 - 2.02 | 0.15 |
|  | rs2036108 A/A | -0.62 | -5.03 - 3.79 | 0.79 |  | rs4680 A/A | -16.98 | -29.3 - -4.67 | 0.007* |
|  |  |  |  |  |  | rs6269 A/G | -2.57 | -8.27 - 3.14 | 0.38 |
| SLC6A2 SNPs | |  |  |  |  | rs6269 G/G | -18.02 | -33.32 - -2.73 | 0.03* |
|  | rs2242446 T/C | 0.86 | -3.54 - 5.26 | 0.70 |  | rs4633 C/T | 5.76 | -1.71 - 13.23 | 0.13 |
|  | rs2242446 C/C | -1.70 | -6.79 - 3.38 | 0.51 |  | rs4633 T/T | 17.33 | 5.18 - 29.48 | 0.01 |
|  | rs36024 T/C | -1.61 | -4.18 - 0.96 | 0.22 |  | rs4818 C/G | 1.02 | -4.45 - 6.48 | 0.72 |
|  | rs36024 C/C | 2.10 | -2.57 - 6.77 | 0.38 |  | rs4818 G/G | 18.91 | 4.13 - 33.69 | 0.02* |
|  | rs1532701 A/G | -0.81 | -3.15 - 1.54 | 0.50 |  | rs165774 G/A | -0.29 | -2.06 - 1.47 | 0.75 |
|  | rs1532701 G/G | -0.42 | -4.81 - 3.97 | 0.85 |  | rs165774 A/A | 0.42 | -2.51 - 3.34 | 0.78 |
|  | rs13333066 C/T | 2.05 | -2.08 - 6.17 | 0.33 |  |  |  |  |  |
|  | rs13333066 T/T | 0.32 | -5.96 - 6.61 | 0.92 | SLC6A3 SNPs | |  |  |  |
|  | rs187714 C/T | -0.34 | -2.72 - 2.04 | 0.78 |  | rs1801253 C/G | -0.32 | -1.92 - 1.27 | 0.69 |
|  | rs187714 T/T | -1.31 | -5.45 - 2.83 | 0.54 |  | rs1801253 G/G | 0.33 | -5.44 - 6.11 | 0.91 |
|  |  |  |  |  |  |  |  |  |  |
| MAOA SNPs | |  |  |  | Treatment (compared to SSRIs) | | |  |  |
|  | rs6323 T/G | -0.82 | -5.19 - 3.55 | 0.72 |  | TCAs | 2.98 | 1.00 - 4.97 | 0.004* |
|  | rs6323 G/G | -6.60 | -14.84 - 1.64 | 0.12 |  | SNRIs | 0.45 | -2.15 - 3.05 | 0.74 |
|  | rs1137070 C/T | 1.84 | -2.52 - 6.2 | 0.41 |  | NaSSAs | 1.08 | -1.94 - 4.1 | 0.48 |
|  | rs1137070 T/T | 6.58 | -1.2 - 14.36 | 0.10 |  | Agomelatine | 2.55 | -0.34 - 5.45 | 0.09 |
|  |  |  |  |  |  |  |  |  |  |
|  |  |  |  |  |  |  |  | R-squared |  |
|  |  |  |  |  |  |  |  | 0.25 |  |

Data is presented as regression coefficients (B), 95% confidence intervals (CI) and total explained variance (r2); * p < 0.05; Significance for p values after correction: **p < 0.0031; *** p < 0.001; HAMD, Hamilton Depression Score Rating Difference; TCAs, tricyclic antidepressants; SNRIs, serotonin–norepinephrine reuptake inhibitors; NaSSAs, noradrenergic and specific serotonergic antidepressants.

**Supplementary Table 4** - Multiple linear regression of total depression cohort covariates (age, gender, diagnosis, type of antidepressant, selected adrenergic genotypes) for the second two week study period (2 to 4 weeks).

| Baseline Predictors | | B | 95% CI | p-value | Baseline Predictors | | B | 95% CI | p-value |
| --- | --- | --- | --- | --- | --- | --- | --- | --- | --- |
| (Constant) | | 2.84 | -2.1 - 7.79 |  |  |  |  |  |  |
| Age | | 2.49 | 0.22 - 4.77 | 0.032* |  |  |  |  |  |
| Gender | | 0.04 | -0.03 - 0.12 | 0.24 |  |  |  |  |  |
| Diagnosis | | -0.04 | -1.65 - 1.58 | 0.97 |  |  |  |  |  |
|  |  |  |  |  |  |  |  |  |  |
| ADRA1A SNPs | |  |  |  | COMT SNPs | |  |  |  |
|  | rs2036108 G/A | 0.60 | -1.04 - 2.24 | 0.47 |  | rs4680 G/A | 6.15 | -1.79 - 14.08 | 0.13 |
|  | rs2036108 A/A | -0.62 | -5.1 - 3.87 | 0.79 |  | rs4680 A/A | 8.22 | -4.32 - 20.75 | 0.20 |
|  |  |  |  |  |  | rs6269 A/G | -0.24 | -6.05 - 5.56 | 0.94 |
| SLC6A2 SNPs | |  |  |  |  | rs6269 G/G | 13.03 | -2.54 - 28.6 | 0.10 |
|  | rs2242446 T/C | -0.90 | -5.37 - 3.58 | 0.70 |  | rs4633 C/T | -6.04 | -13.64 - 1.56 | 0.12 |
|  | rs2242446 C/C | -2.23 | -7.4 - 2.94 | 0.40 |  | rs4633 T/T | -8.74 | -21.1 - 3.62 | 0.17 |
|  | rs36024 T/C | -0.08 | -2.69 - 2.54 | 0.96 |  | rs4818 C/G | 0.72 | -4.84 - 6.28 | 0.80 |
|  | rs36024 C/C | 1.94 | -2.81 - 6.69 | 0.42 |  | rs4818 G/G | -11.75 | -26.79 - 3.29 | 0.13 |
|  | rs1532701 A/G | 2.77 | 0.4 - 5.16 | 0.023* |  | rs165774 G/A | -0.05 | -1.84 - 1.75 | 0.96 |
|  | rs1532701 G/G | 7.07 | 2.61 - 11.54 | 0.002** |  | rs165774 A/A | -2.09 | -5.07 - 0.88 | 0.17 |
|  | rs13333066 C/T | -0.68 | -4.87 - 3.52 | 0.76 |  |  |  |  |  |
|  | rs13333066 T/T | -2.85 | -9.24 - 3.55 | 0.38 | SLC6A3 SNPs | |  |  |  |
|  | rs187714 C/T | -0.01 | -2.43 - 2.41 | 0.99 |  | rs1801253 C/G | -0.29 | -1.91 - 1.33 | 0.73 |
|  | rs187714 T/T | -4.18 | -8.39 - 0.03 | 0.06 |  | rs1801253 G/G | -2.60 | -8.47 - 3.28 | 0.39 |
|  |  |  |  |  |  |  |  |  |  |
| MAOA SNPs | |  |  |  | Treatment (compared to SSRIs) | | | |  |
|  | rs6323 T/G | 1.28 | -3.17 - 5.72 | 0.58 |  | TCAs | 1.42 | -0.6 - 3.44 | 0.17 |
|  | rs6323 G/G | 1.79 | -6.6 - 10.17 | 0.68 |  | SNRIs | -0.10 | -2.75 - 2.54 | 0.94 |
|  | rs1137070 C/T | -1.67 | -6.11 - 2.76 | 0.46 |  | NaSSAs | -2.48 | -5.55 - 0.59 | 0.12 |
|  | rs1137070 T/T | -1.22 | -9.14 - 6.7 | 0.77 |  | Agomelatine | -1.67 | -4.61 - 1.27 | 0.27 |
|  |  |  |  |  |  |  |  |  |  |
|  |  |  |  |  |  |  |  | R-squared |  |
|  |  |  |  |  |  |  |  | 0.25 |  |

Data is presented as regression coefficients (B), 95% confidence intervals (CI) and total explained variance (r2); * p < 0.05; Significance for p values after correction: **p < 0.0031; *** p < 0.001; HAMD, Hamilton Depression Score Rating Difference; TCAs, tricyclic antidepressants; SNRIs, serotonin–norepinephrine reuptake inhibitors; NaSSAs, noradrenergic and specific serotonergic antidepressants.
